# Comprehensive analysis to identify a novel PTEN-associated ceRNA regulatory network as a prognostic biomarker for lung adenocarcinoma

**DOI:** 10.3389/fonc.2022.923026

**Published:** 2022-08-24

**Authors:** Rui Xin, Biao Shen, Ying-Jie Jiang, Ji-Bin Liu, Sha Li, Li-Kun Hou, Wei Wu, Cheng-You Jia, Chun-Yan Wu, Da Fu, Yu-Shui Ma, Geng-Xi Jiang

**Affiliations:** ^1^ Department of Nuclear Medicine, Shanghai Tenth People’s Hospital, Tongji University School of Medicine, Shanghai, China; ^2^ Department of Thoracic Surgery, Affiliated Tumor Hospital of Nantong University, Nantong, China; ^3^ Department of Pathology, Navy Military Medical University Affiliated Changhai Hospital, Shanghai, China; ^4^ Institute of Oncology, Affiliated Tumor Hospital of Nantong University, Nantong, Jiangsu, China; ^5^ Department of Pathology, Shanghai Pulmonary Hospital, Tongji University School of Medicine, Shanghai, China; ^6^ Institute of Pancreatic Diseases, Ruijin Hospital, Shanghai Jiaotong University School of Medicine, Shanghai, China; ^7^ Cancer Institute, Longhua Hospital, Shanghai University of Traditional Chinese Medicine, Shanghai, China; ^8^ Department of Thoracic Surgery, Navy Military Medical University Affiliated Changhai Hospital, Shanghai, China

**Keywords:** ceRNA network, LINC00460, miR-150-3p, LUAD, PTEN

## Abstract

Lung adenocarcinoma (LUAD) is one of the most prevalent forms of lung cancer. Competitive endogenous RNA (ceRNA) plays an important role in the pathogenesis of lung cancer. Phosphatase and tensin homolog (PTEN) is one of the most frequently deleted tumour suppressor genes in LUAD. The present study aimed to identify a novel PTEN-associated-ceRNA regulatory network and identify potential prognostic markers associated with LUAD. Transcriptome sequencing profiles of 533 patients with LUAD were obtained from TCGA database, and differentially expressed genes (DEGs) were screened in LUAD samples with PTEN high- (PTEN^high^) and low- (PTEN^low^) expression. Eventually, an important PTEN-related marker was identified, namely, the LINC00460/miR-150-3p axis. Furthermore, the predicted target genes (EME1/HNRNPAB/PLAUR/SEMA3A) were closely related to overall survival and prognosis. The LINC00460/miR-150-3p axis was identified as a clinical prognostic factor through Cox regression analysis. Methylation analyses suggested that abnormal regulation of the predicted target genes might be caused by hypomethylation. Furthermore, immune infiltration analysis showed that the LINC00460/miR-150-3p axis could alter the levels of immune infiltration in the tumour immune microenvironment, and promote the clinical progression of LUAD. To specifically induce PTEN deletion in the lungs, we constructed an STP mouse model (SFTPC-rtTA/tetO-cre/Pten^flox/+^). Quantitative PCR (qPCR) and immunohistochemical (IHC) analysis were used to detect predicted target genes. Therefore, we revealed that the PTEN-related LINC00460/miR-150-3p axis based on ceRNA mechanism plays an important role in the development of LUAD and provides a new direction and theoretical basis for its targeted therapy.

## Introduction

Lung cancer is one of the most life-threatening cancers in the world (1). Approximately 40% of the diagnosed lung cancer cases are lung adenocarcinoma (LUAD), whose morbidity and mortality have increased recently (2). The etiology of LUAD is very complex and involves genetic, environmental, and behavioral factors (3). LUAD has become a global concern owing to its high recurrence and metastasis rate. The prognosis of patients with LUAD is poor, and 5-year survival rate is as low as 16% (4). In addition, most patients with lung cancer do not have a clearly defined drug target (5). Current research on LUAD is mainly focused on predicting new non-invasive molecular targets (6), which is necessary to improve LUAD treatment and chemoprevention.

Phosphatase and tensin homolog (PTEN) is a lipid phosphatase (7). PTEN is an important tumour suppressor (8), which plays an important role in lung cancer development (9). Loss of PTEN expression is relatively common in LUAD and can be attributed to epigenetic and post-transcriptional mechanisms (10). However, the regulatory mechanisms underlying the loss of PTEN expression in lung cancer remain unknown (11). In recent years, scholars have developed great interest in the competitive endogenous RNA (ceRNA) network hypothesis, which reveals a new mechanism of RNA interaction (12). Studies have reported the effects of dysregulation of ceRNA expression on the pathogenicity and progression of cancer (13). Because PTEN is a powerful tumour suppressor gene, even subtle changes in its protein expression may have a profound impact on the incidence and invasiveness of tumors (14). Therefore, the association between ceRNA and PTEN is particularly important and has been extensively studied (15). Predicting the future, it is important to identify new targeted drugs that can be used to treat patients with LUAD with PTEN mutations.

The Cancer Genome Atlas (TCGA) uses innovative genome analysis techniques to accelerate the comprehensive understanding of cancer genetics and help in developing new cancer therapies, diagnostic techniques, and prevention strategies (16). Genome sequencing data for various types of cancers have been annotated in the TCGA database (17). Long non-coding RNAs (lncRNAs) can regulate the expression of targeted messenger RNAs (mRNAs) by competing with microRNAs (miRNAs) for binding (18). Based on the ceRNA hypothesis, TCGA database can be used to construct a novel ceRNA network associated with LUAD to develop new diagnostic and prognostic biomarkers (19).

In this study, we screened for DEGs in patients with LUAD using data from TCGA and attempted to build a novel ceRNA regulatory network related to PTEN. Firstly, the differential expression of PTEN (PTENhigh [n = 266] and PTENlow [n = 267] based on the median PTEN expression) was analyzed in 533 LUAD samples, and in total 298 DElncRNAs, 188 DEmiRNAs and 1527 DEmRNAs were obtained. Subsequently, all differentially expressed RNAs (DERNAs) were analyzed via expression level, overall survival, correlation, and nuclear–cytoplasmic localization analysis. Finally, the mir-150-3p–LINC00460–EME1/HNRNPAB/PLAUR/SEMA3A axis was identified as a clinical prognostic marker. In addition, methylation analysis showed that abnormal regulation of HNRNPAB and PLAUR may be caused by hypomethylation. Immune infiltration analysis showed that LINC00460/miR-150-3p could alter the levels of immune infiltration in the tumour immune microenvironment and promote the clinical progression of LUAD. Most importantly, we elucidated the expression of predicted target genes in PTEN-deficient lung tissues.

**Figure 1:**
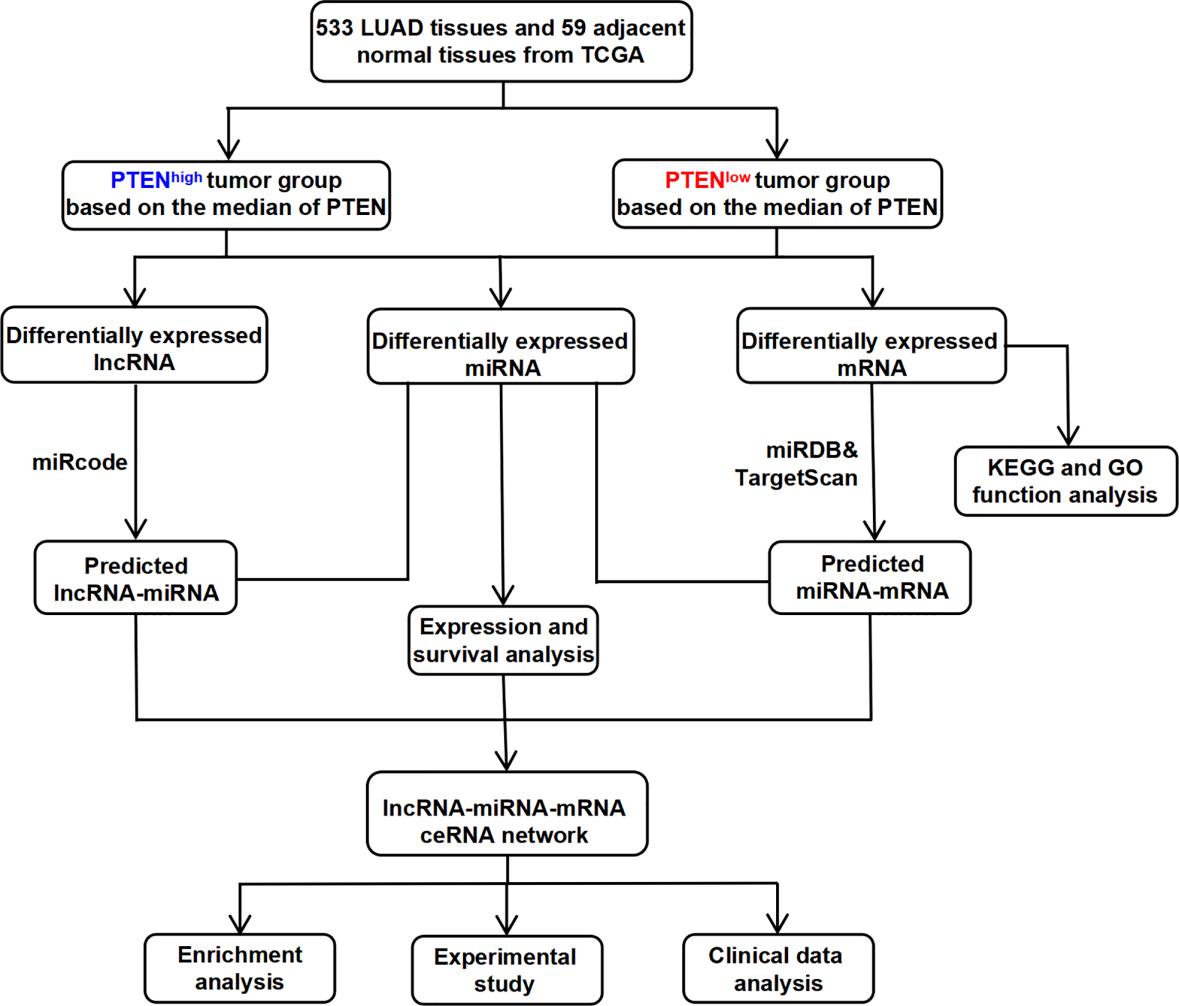
The flow chart for the construction process of the novel PTEN-related ceRNA regulatory network in LUAD.

Therefore, this study aimed to identify rich functional pathways, analyze patient survival, and screen for potential DEGs to identify innovative and reliable prognostic biomarkers associated with the development of LUAD. Therefore, the present study helps to understanding the molecular mechanism of LUAD development and may provide novel ideas for clinical prediction and treatment (**Figure 1**).

## Materials and methods

### Data processing and analysis

The sequencing data and clinical information of 533 lncRNAs/mRNAs and 505 miRNAs of patients with LUAD were downloaded from TCGA website (https://portal.gdc.cancer.gov) (20). All raw RNA-Seq data (lncRNA, miRNA, and mRNA) were normalized as fragments per kilobase of exon model per million mapped fragment reads. The starBase v2.0 database (http://starbase.sysu.edu.cn) was used to convert miRNA sequences to human mature miRNA names (21). The Encyclopedia of Cancer Cell Lines (CCLE, https://portals.broadinstitute.org/ccle) (22) and Human Protein Atlas (HPA, http://www.proteinatlas.org) (23) were used to test the cell lines and protein levels, respectively. The cBioPortal (http://www.cbioportal.org) was used to obtain the gene mutation status.

All LUAD samples were classified as PTENhigh and PTENlow (median expression levels of PTEN). DEmRNAs, DElncRNAs, and DEmiRNAs were screened by comparing PTENhigh (n=266) and PTENlow (n=267) tumour samples. The screening thresholds of DEmRNAs, DElncRNAs, and DEmiRNAs were |logFC| > 0.5 and P < 0.05. Volcano maps were drawn using the GraphPad Prism software (version 8.4). Heatmaps were drawn using the TBtools software (version 0.655).

### Construction and identification of the ceRNA network

We constructed the ceRNA network by the following steps: (1) the miRcode database (http://www.mircode.org) was used to identify DEmiRNAs which interacted with DElncRNAs (24). (2) The miRDB (http://www.mirdb.org) and TargetScan (http://www.targetscan.org) databases were used to predict the target genes of interacted DEmiRNAs and construct miRNA–mRNA interaction pairs (25). (3) The ‘Venn Diagram’ package of the ‘R’ software was used to select target genes that duplicated with DEmRNAs. (4) The LNCipedia database (https://lncipedia.org) was used to obtain DElncRNAs sequences. The lncLocator database (http://www.csbio.sjtu.edu.cn/bioinf/lncLocator) was used to identify the cell location of DElncRNAs (26). (5) Finally, the ceRNA networks were visualized using the Cytoscape software (http://www.cytoscape.org) (27). (6) We visualized the ‘pathways’ through bubble graphs and presented the KEGG analysis results using the R software package ‘ggplot2’. The Cytoscape plug-in ‘cytohubba’ was used to identify a hub triple regulatory network (top 100 hub ceRNAs).

### Functional enrichment analysis of DEmRNAs

To investigate the pathways associated with the screened LUAD prognostic markers, the top 100 genes associated with target genes were obtained from GEPIA (http://gepia.cancer-pku.cn) (28). We used the Gene Ontology (GO) and Kyoto Encyclopedia of Genes and Genomes (KEGG) pathways on Metascape (http://metascape.org) website to analyze the enrichment function of DEmRNAs in the triple regulatory network, including biological processes (BP), cellular components (CCs), molecular functions (MFs) and KEGG pathways (29). The pathways visualized on bubble graphs using the R package ‘ggplot2’ to present the pictures of KEGG and GO analysis. The protein–protein interaction (PPI) interaction networks and interacting genes were predicted with the STRING database (https://string-db.org) (30) and GeneMania database (http://genemania.org) (31).

### Methylation and expression analysis

Studies showed that DNA methylation is a significant epigenetic mechanism, which is able to regulate gene expression and influence the behavior of cancer cells (32). The UALCAN (http://ualcan.path.uab.edu) was used to analyze the degree of methylation of target genes in LUAD. The MethSurv (https://biit.cs.ut.ee/methsurv) was used to obtain the CpG methylation data of target genes, which contain important information regarding a single CpG island. MEXPRESS (https://mexpress.be) was used for visualizing TCGA and methylation expression and clinical information (33).

### Mutation analysis

The cBioPortal database contains multiple types of data such as somatic mutations, DNA methylation, protein enrichment, and miRNA expression to facilitate the study of multidimensional cancer gene datasets. The mutations of target genes in LUAD were visualized using cBioPortal.

### Immune infiltration level and expression analysis

To investigate the association of the expression of target genes and tumor infiltrating immune cells, we applied TIMER2.0 (http://timer.cistrome.org), which is an online tool for the analysis and visualization of the correlation between immune infiltrate levels and a number of variables across diverse cancer types. The top 20 genes (PCC/Pearson’s r > 0.4) associated with target genes were obtained from GEPIA (http://gepia.cancer-pku.cn). GEPIA databases were used to analyze and visualize the level of immune infiltration. We estimated the correlations between target genes and the abundance of tumour-infiltrating immune cells, the prognostic value of DERNAs, and the copy number of target genes.

### Animal model and genotyping

All experimental procedures were approved by the Institutional Animal Care and Use Committee (IACUC) guidelines at Tongji University School of Medicine (SYDW-19-215). SFTPC-rtTA mice (Jackson Laboratory; Sp-c-rtTA; 006225) were crossed with tetO-cre transgenic mice (Jackson Laboratory; (tetO)7-Cre; 006224) to obtain age-matched SFTPC-rtTA/tetO-cre mice for experiments. We crossed conditionally PTEN-null mice (Southern Biotech; PTENflox/+, NM-18004) with SFTPC-rtTA/tetO-cre mice and generated lung epithelial-specific PTEN conditionally knockout mice (STP mice), which were intraperitoneally injected with doxycycline (5 mg/kg) thrice a week for 1 month. All mice were housed in standard cages (6 mice/cage) with ad libitum access to food and water at a temperature of 22°C–26°C and relative humidity of 35%–55%.

Polymerase chain reaction (PCR) genotyping was performed using One step mouse genotyping kit (Vazyme, PD101). PTEN forward primer (5′- CAAGCACTCTGCGAACTGAG-3′) and PTEN reverse primer (5′- AAGTTTTTGAAGGCAAGATGC-3′), and performed on a S1000™ Thermal Cycler (Bio-Rad): 94°C for 5 min, 94°C for 20 s, 55°C for 30 s, 72°C for 30s (35 times in step 2); 72°C for 5 min, and a final hold at 12°C. PCR products were run in 1.5% agarose gel (SFTPC: Heterozygote = 450 bp and 297 bp; tetO-Cre: Heterozygote = 324 bp and 100 bp; PTEN: Heterozygote = 328 bp and 156 bp).

### RNA extraction and RT-PCR

Total RNA was extracted using the TRIzol reagent and reverse transcribed to cDNA using the PrimeScript™ RT reagent Kit (Takara, RR047A) kit. The synthesized cDNA was used as a template for real-time polymerase chain reaction (RT-PCR) and performed on a CFX96 Real-Time system (Bio-Rad) using SYBR®Premix Ex Taq™ (Takara, RR420A) kit. PCR primers for mouse genes (EME1, HNRNPAB, PLAUR, and SEMA3A) were designed by Sangon Biotech. The relative fold change in the expression of target genes normalized to the expression of the corresponding control was calculated using the comparative Ct method. The primers are listed in **Table S2**.

### HE staining and immunohistochemical (IHC) analysis

The sections of lung tissues were used for HE staining as well as immunohistochemical analysis. The morphological changes and expressions of HNRNPAB/PLAUR and SEMA3A in STP mice lung tissues and human lung cancer tissues were analyzed. HNRNPAB antibody (Rabbit Polyclonal Antibody, D264202, sangon,1:40); PLAUR antibody (Rabbit Polyclonal Antibody, D121140, sangon, 1:100) and SEMA3A antibody (Rabbit Polyclonal Antibody, Abp53467, abbkine, 1:100) were used for all tissue sections. Each section underwent specific antibody detection. The images were taken using an inverted Nikon Eclipse TS2R (Original magnification: ×200).

### Targeted drug analysis

The HERB database (http://herb.ac.cn) is a database of traditional Chinese medicine, which is used to query possible targeted herbs. The cancer analysis platform GSCA (http://bioinfo.life.hust.edu.cn/web/GSCALite) was used to integrate the correlations between drugs and target genes in GDSC and CTRP databases.

### Statistics

The GraphPad Prism (version 8.4) and SPSS 23.0 software were used to compare the differential expression. The results of the correlation and survival analysis of the lncRNA–miRNA–mRNA network was expressed as median and 95% CI. The Mann–Whitney test and independent t-test were used to calculate differences between the two groups of data. One-way analysis of variance with the chi-square test was used to evaluate the difference between different groups. A P-value < 0.05 was considered statistically significant.

## Results

### The role of PTEN down expression in LUAD patients

**Figure 2:**
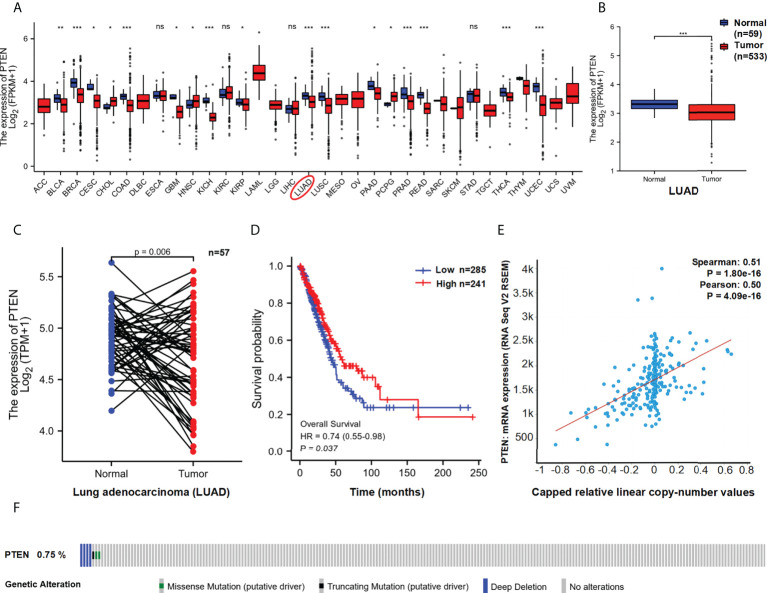
Functional characteristics of PTEN in LUAD. **(A)** PTEN expression in pan-cancer and **(B)** LUAD paired samples. The expression **(C)** and overall survival **(D)** of PTEN in LUAD (Low=285, High=241). **(E)** Correlation between PTEN copy number and expression. **(F)** The distribution of PTEN genome changes in TCGA. ns, no significant; *p < 0.05; **p < 0.01; ***p < 0.001.

The possible function of PTEN in LUAD based on TCGA data was investigated and found that PTEN expression was significantly lower in LUAD tissues than in normal tissues (P < 0.01) (**Figure 2A** and **Supplemental Figures 1A, B**). Similar results were observed in the paired control and LUAD samples in TCGA database (normal = 57, tumour = 57) (P=0.006) (**Figure 2B**). The expression level of PTEN total protein in lung cancer is shown in **Figure S1C**. The HPA database contains protein expression data in various human tissues (34). IHC staining obtained from the HPA database also confirmed the differential PTEN expression (**Figure S1D** and **Supplemental Table 1**).

Furthermore, we used TCGA data to assess the correlation between PTEN expression and prognosis among patients with LUAD. As shown in **Figure 2C**, lower expression of PTEN was associated with poor overall survival (OS) (P = 0.037). In addition, we analysis the genomic alterations and copy number of PTEN. A positive correlation was found between PTEN copy number and mRNA expression in LUAD samples (R > 0.5, P < 0.01) (**Figure 2D**). The distribution of genomic changes in PTEN (0.75% deep deletion) in LUAD samples is shown in **Figure 2E**. In conclusion, PTEN deletion may be one of the main mechanisms of LUAD.

### Identification of PTEN-related DEGs in LUAD

**Figure 3:**
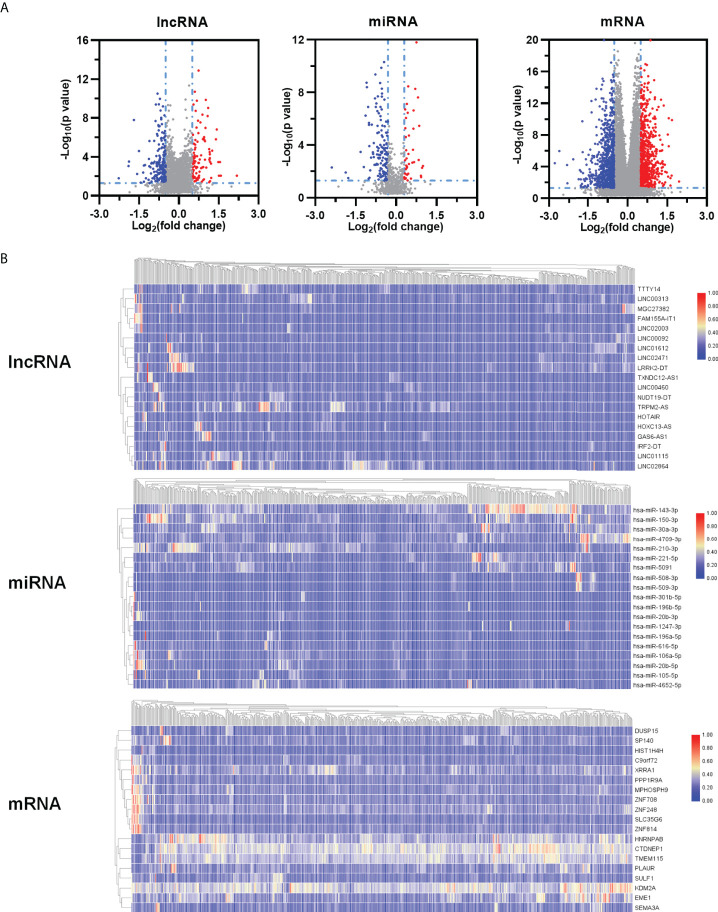
Identification of DEGs. **(A)** Volcano maps and heatmaps **(B)** of differently expressed ceRNAs between PTEN^high^ and PTEN^low^ groups in LUAD.

**Figure 4:**
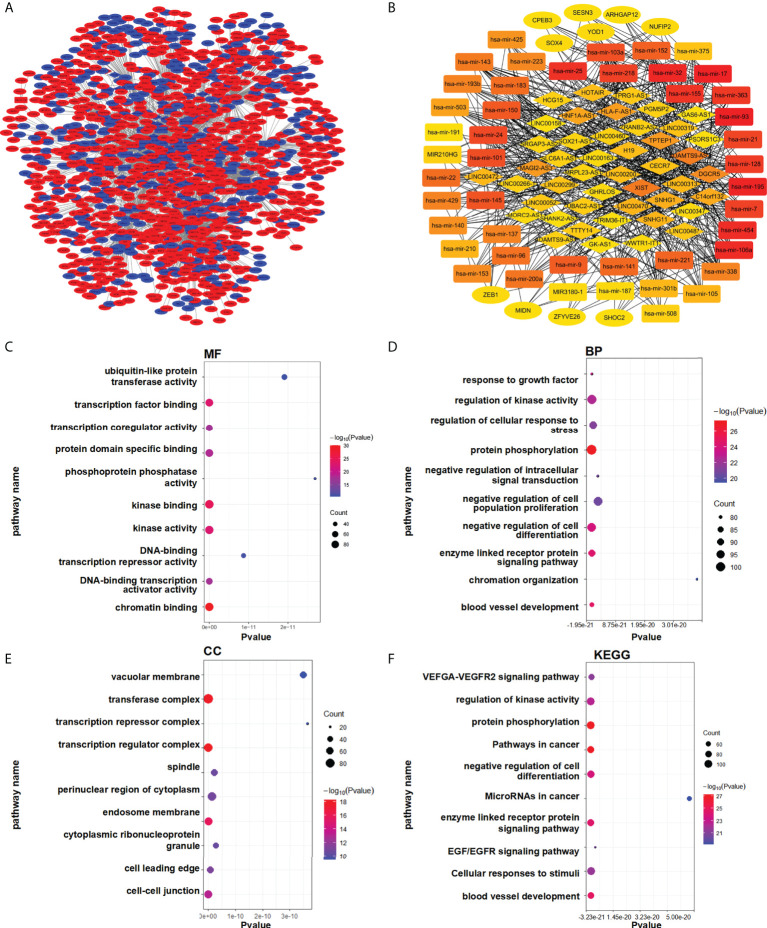
Enrichment analysis of the regulatory network. **(A)** Triple ceRNA network. **(B)** Top 100 hub ceRNAs in the network. **(C–F)** Functional enrichment analysis of the DEmRNAs. **(C)** MF, **(D)** BP, **(E)** CC, and **(F)** KEGG.

We speculated that a PTEN related ceRNA network could serve as a potential prognostic model for LUAD. To verify this hypothesis, we first downloaded all data of LUAD patients from TCGA database and divided into two groups (PTENhigh and PTENlow) based on the median of PTEN expression level. Subsequently, we screened differentially expressed lncRNAs, miRNAs, and mRNAs according to the criteria of |logFC| > 0.5 and P < 0.05. Eventually, 298 DElncRNAs (112 upregulated and 186 downregulated), 188 DEmiRNAs (43 upregulated and 145 downregulated), and 1527 DEmRNAs (718 upregulated and 809 downregulated) were obtained (**Figure 3A**). The heatmap was generated to demonstrate the 15 important DElncRNAs, DEmiRNAs, and DEmRNAs (**Figure 3B**). PTEN-related DElncRNA–DEmiRNA–DEmRNA triple regulatory networks were constructed by Cytoscape 3.7 (**Figure 4A**). The central triple regulatory network was screened and the top 100 most relevant DERNAs were identified by Cytoscape plug-in ‘cytohubba’ (**Figure 4B**).

For analysis potential biological functions related to the network in LUAD, we used the Metascape database to assess the functions of all DEmRNAs via GO and KEGG pathway analyses (**Figure 4C**). The results showed that DEmRNAs participating in the networks were enriched in pathways associated with ‘cancer’ and ‘EGFR signaling’, which are related to lung cancer (**Figure 4D–F**).

### Construction of the triple regulatory network

LncRNAs and mRNAs with the common miRNA target site could form complex networks of interactions, usually called the competing endogenous RNA (ceRNA) network (35). We attempt to establish a novel lncRNA-mRNA-miRNA ceRNA network from the DERNAs of LUAD. We combined the OS of LUAD patients to comprehensively analysis the high- and low-PTEN expression groups. Through the miRcode database, 44 predicted miRNAs that potentially target DElncRNAs were identified. Only 20 out of the predicted miRNAs were selected after taking the intersection with 188 DEmiRNAs.

Next, we used miRDB and TargetScan databases to identify downstream target mRNAs through these 20 miRNAs. The results showed that 1219 mRNAs were identified. Intersect these mRNAs with all DEmRNAs, only 240 predicted mRNAs remained. Finally, OS analysis of patients showed a total of 12 DElncRNAs (AC007389.1, AC011374.1, AC078802.1, AL391152.1, C10orf25, GRIK1-AS1, HMGA1P4, LINC00322, LINC00460, MAGI2-AS3, SFTA1P and ZNF503-AS1), 2 DEmiRNAs (hsa-mir-150-3p and hsa-mir-200a-3p) and 10 DEmRNAs (ARRDC3, CNR2, EME1, HNRNPAB, PAQR4, PLAUR, SEMA3A, ZEB2, ZNF41 and ZWINT) were significantly related to prognosis in LUAD (**Figure S2**).

**Figure 5:**
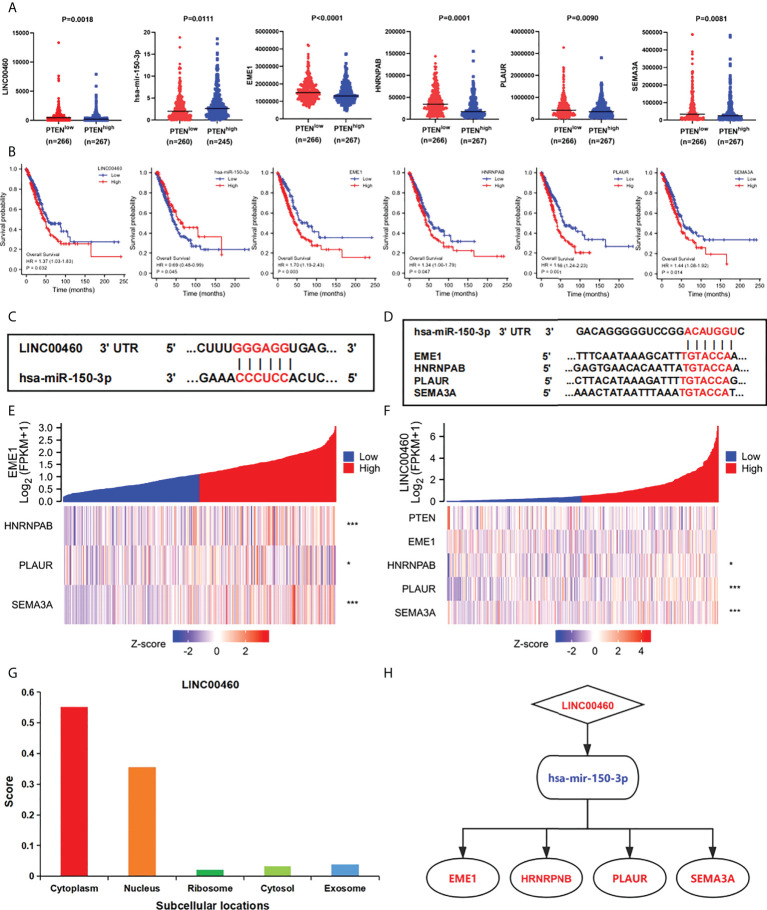
Construction of the triple regulatory network. **(A)** The expression of ceRNAs in PTEN^high^ and PTEN^low^ groups. **(B)** The OS of ceRNAs in the network. **(C)** Predictive binding location between miRNA and lncRNA. **(D)** Predictive binding location between lncRNA and mRNA. **(E)** Correlation analysis between predictive genes in LUAD. **(F)** Correlation analysis between predictive ceRNAs and PTEN in LUAD. **(G)** The cellular localization of LINC00460. **(H)** The predicted triple regulatory network in LUAD. *p < 0.05; ***p < 0.001.

To construct the prognostic model of PTEN expression-related LUAD, we further analysis the expression of DERNAs. The results revealed that hsa-mir-150-3p was upregulated, whereas LINC00460 was downregulated and four mRNAs (EME1, HNRNPAB, PLAUR and SEMA3A) were downregulated in PTENhigh groups (P < 0.05) (**Figure 5A**). OS analysis showed 1 DElncRNA (LINC00460), 1 DEmiRNA (miR-150-3p), and 4 DEmRNAs (EME1/HNRNPAB/PLAUR and SEMA3A) were found to be related to prognosis (**Figure 5B**). The 3’ UTR binding locations of LINC00460, hsa-miR-150, and EME1/HNRNPAB/PLAUR/SEMA3A identified using the miRDB and TargetScan databases are shown in **Figures 5C, D**.

In addition, through expression correlation analysis, we analysis the correlation between predictive ceRNA and PTEN in LUAD samples. We discovered that a positive relationship between LINC00460 expression and HNRNPAB/PLAUR/SEMA3A expression (P < 0.05) (**Figures 5E, F**). Furthermore, negative correlations between PTEN and LINC00460/EME1/HNRNPAB/PLAUR expressions were observed. Most importantly, the expression levels between predicted genes were significantly correlated. (P < 0.05) (**Figure S14**). Finally, we analysis the subcellular location of DElncRNAs using the lncLocator and found that LINC00460 was located in the cytoplasm (**Figure 5G**). Therefore, we established a PTEN-associated lncRNA–mRNA–miRNA ceRNA network based on DERNAs in LUAD samples (**Figure 5H**).

### Expression of ceRNA in LUAD

**Figure 6:**
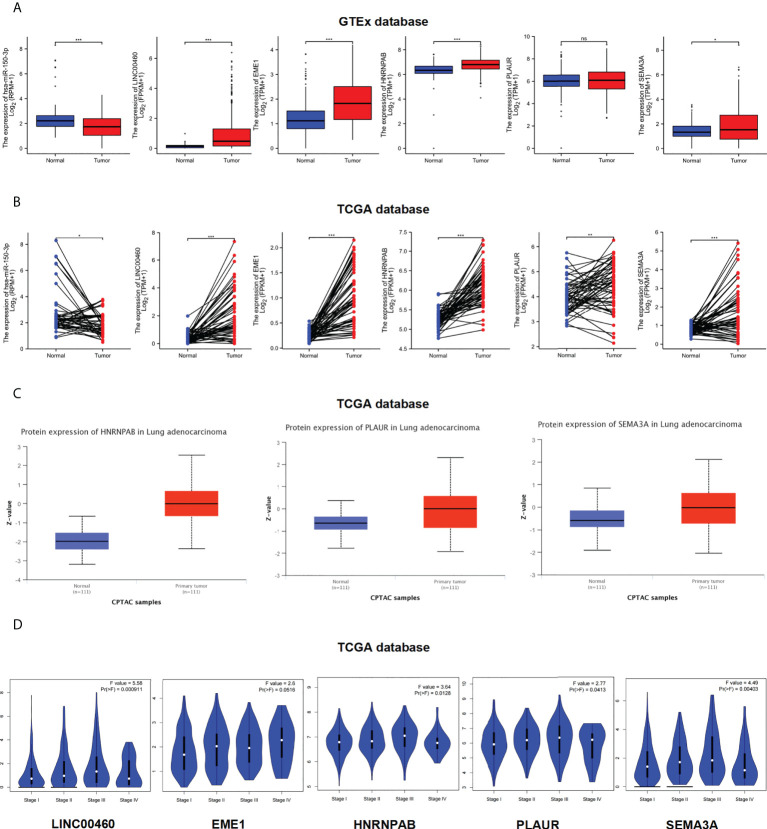
Expression level of ceRNAs. **(A–C)** Expression levels of ceRNAs in LUAD paired samples (normal = 57, tumor = 57). **(A)** GTEx database. **(B)** TCGA database. **(C)** CPTAC database. **(D)** Clinical pathological stages of ceRNAs. ns, no significant; *p < 0.05; **p < 0.01; ***p < 0.001.

To examine the potential functions of the identified ceRNAs, we analysis the expression of LINC00460/mir-150-3p/EME1/HNRNPAB/PLAUR and SEMA3A in LUAD and normal samples using TCGA, GTEx and CPTAC databases. The expression of LINC00460/EME1/HNRNPAB/SEMA3A was significantly higher in LUAD tissues than in the corresponding normal tissues (**Figure 6A**). In paired tissues (normal = 57, tumour = 57), the expression of PLAUR was also significantly different (P < 0.01) (**Figure 6B**). The expression of mir-150-3p was significantly lower in LUAD tissues than in the corresponding normal tissues (P < 0.001) (**Figure 6A**). In the CPTAC dataset, the expression of HNRNPAB/PLAUR/SEMA3A total protein was higher in the primary tissues of LUAD (P < 0.001) than in normal tissues (**Figure 6C**). Similar data for EME1 was not available in the CPTAC dataset.

Furthermore, we used the ‘Stage Plot’ module of GEPIA database to observe the correlation between gene expression and pathological stages in LUAD. The pathological stages of LUAD were significantly correlated with the expression of LINC00460/HNRNPAB/PLAUR/SEMA3A but not with the expression of EME1 (**Figure 6D**). We used TCGA database to analysis the expression of ceRNAs across various cancer types. As shown in **Figure S3**, **S4**, the expression of LINC00460/EME1/HNRNPAB/PLAUR and SEMA3A was higher in most tumour tissues, including LUAD, than in the corresponding control tissues (P < 0.001). However, the expression of mir-150-3p was lower in most pan-cancer tissues (P < 0.05).

### Enrichment analysis and gene/protein interaction

**Figure 7:**
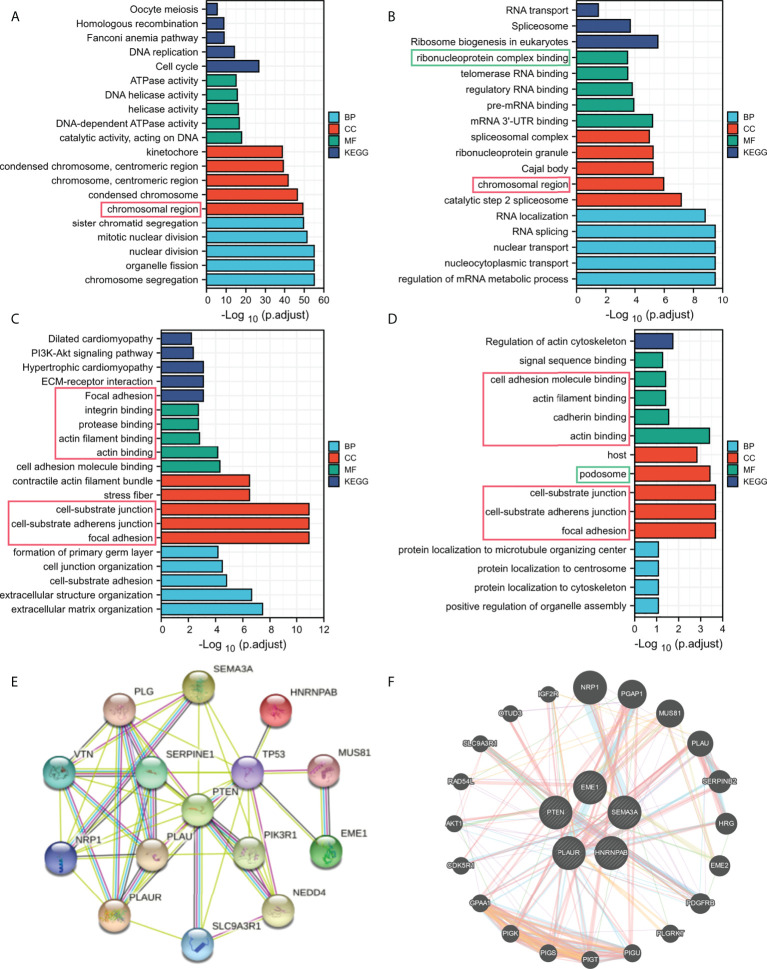
Functional enrichment analysis of the predicted target genes. **(A)** EME1. **(B)** HNRNPAB. **(C)** PLAUR. **(D)** SEMA3A. **(E)** The PPI network map. **(F)** Functional association networks.

Regulation among ceRNAs in interaction networks plays an important role in cancer pathogenesis (36). To identify the potential biological functions of EME1/HNRNPAB/PLAUR/SEMA3A, GO and KEGG analyses were performed on the top 200 most correlated genes (**Figures 7A–D**). Enrichment analysis confirmed that EME1- and HNRNPAB-related genes were enriched in the ‘chromosomal region’ pathway. The results also confirmed that most pathways enriched by PLAUR- and SEMA3A-related genes were duplicated, suggesting that these two genes are involved in the same pathways leading to cancer development. In addition, the results revealed that among the pathways enriched by PTEN-related genes, only two pathways (‘ribonucleoprotein complex binding’ and ‘podosome’) were the same as those 4 predicted target gene enrichment pathways (**Figure S5A**).

The PPI network maps generated using the STRING dataset showed 10 potential target proteins (**Figure 7E**, **Figure S5C–F**). EME1/HNRNPAB/PLAUR/SEMA3A and PTEN were co-expressed with TP53 and other proteins. Furthermore, we used the GeneMANIA dataset to predict the functional association network of EME1/HNRNPAB/PLAUR/SEMA3A and PTEN, which revealed 20 potential target genes (**Figure 7F**, **Figure S5B**). Moreover, we found that EGFR is one of the potential target proteins of PLAUR, which is one of the most common mutation targets in lung cancer (**Figure S8E**).

Finally, we analysis the correlation between the predicted ceRNAs and the mainstream therapeutic targets in LUAD (**Figure S13**). The expression of PLAUR was significantly associated with EGFR expression (R=0.378; P < 0.001). The expression of SEMA3A was significantly associated with EGFR and PD-L1 in LUAD (R=0.300, R=0.352; P < 0.001).

### Clinical relevance of ceRNAs in LUAD

**Figure 8:**
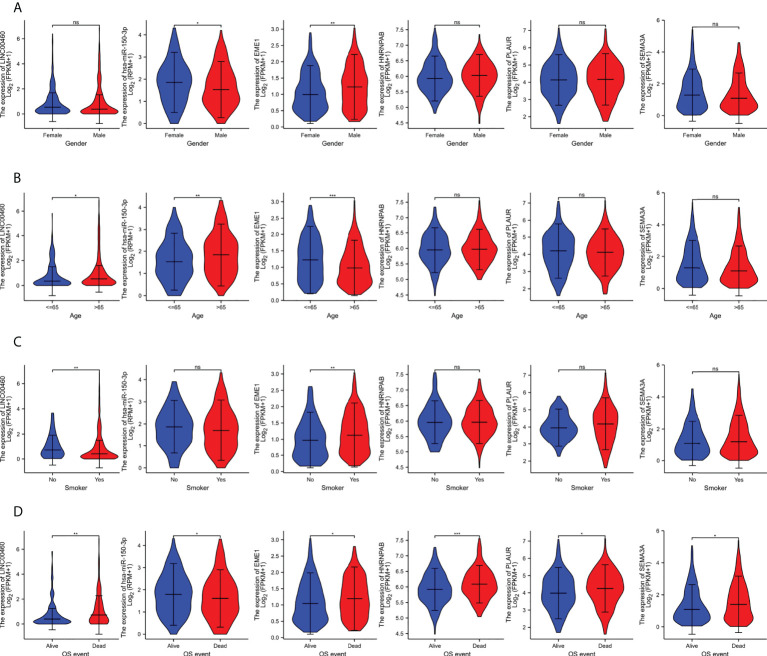
The clinical relevance of predicted target genes in LUAD Patients. **(A)** EME1. **(B)** HNRNPAB. **(C)** PLAUR. **(D)** SEMA3A. ns, no significant; *p < 0.05; **p < 0.01; ***p < 0.001.

We examined the relationship between the expression of DERNAs and clinical features via correlation analysis. The results indicated that patients with high expression of LINC00460/EME1/HNRNPAB/PLAUR/SEMA3A had poor OS (**Figure 8D**). However, the expression of hsa-mir-150-3p in dead cases was lower (P < 0.05), which was consistent with our hypothesis.

In addition, the expression of EME1 was significantly higher in male patients than in female patients (P < 0.01) (**Figure 8A**), in patients aged ≤ 65 years than in those aged > 65 years (**Figure 8B**) and in smokers than in non-smokers (**Figure 8C**). Therefore, the expression of EME1 was significantly associated with ‘gender’, ‘age’ and ‘smoking history of patients’. Similarly, the expression of hsa-mir-150-3p was significantly associated with ‘gender’ and ‘age’. The expression of LINC00460 was significantly associated with the ‘age’ and ‘smoking status of patients’.

To assess whether the identified DERNAs were independent predictors of OS in patients with LUAD, we performed univariate and multivariate Cox regression analyses. In the univariate Cox regression analysis, ‘TNM stage’, ‘lymph node metastasis’, ‘distant metastasis of the tumour’ and expression of EME1/HNRNPAB/PLAUR/SEMA3A were identified as independent risk factors for OS (P < 0.05); in multivariate Cox regression analysis, ‘lymph node metastasis’, ‘distant metastasis of the tumour’ and expression of EME1/PLAUR were identified as independent risk factors for OS (P < 0.05) (**Table S3–S7**).

### Correlation between predicted target gene expression and methylation

**Figure 9:**
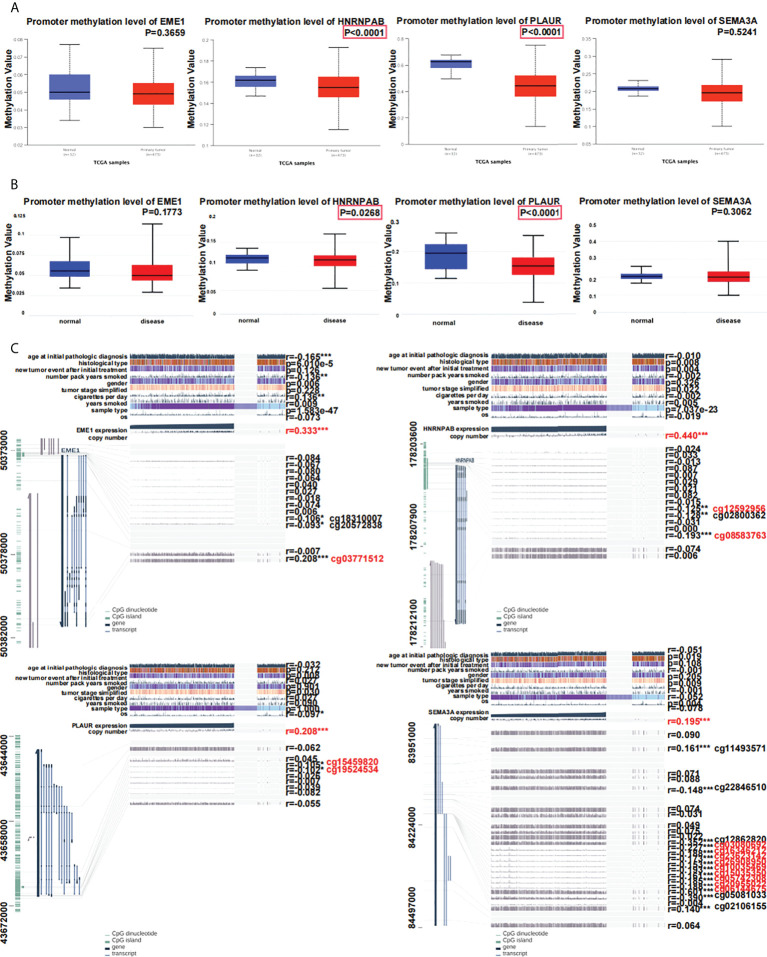
Relationship between predicted target gene expression and methylation. **(A)** Evaluation of methylation on UALCAN. **(B)** Evaluation of methylation with DiseaseMeth.qqqqqqqqqq **(C)** The methylation sites of DNA sequence association with gene expression. *p < 0.05; **p < 0.01; ***p < 0.001.

The UALCAN and DiseaseMeth (version 2.0) databases were used to assess methylation levels (**Figures 9A, B**). According to the MEXPRESS database, the methylation of EME1 was significantly associated with several clinical factors including ‘age at initial pathological diagnosis’, ‘histological type’, ‘number of pack-years’, ‘gender’, ‘cigarettes per day’ and ‘sample type’ (**Figure 9C**). A correlation between the methylation and copy number alterations of EME1/HNRNPAB/PLAUR/SEMA3A was observed in LUAD samples (r = 0.333; 0.440; 0.208 and 0.195, respectively, **Figure 9C**).

Methylation of genes occurs at multiple sites. Methylation of EME1 and PLAUR occurred at cg18310007 and cg20572838 (P < 0.05) (**Figure 9C**). EME1 methylation occurs at the same site of cg03771512 in LUAD, LUSC, and LIHC (**Figure S6A**). Methylation of HNRNPAB occurs at the same site of cg08583763 in LUAD, LUSC, LIHC, and COAD (**Figure S6B**). Methylation of PLAUR occurs at the same site of cg15459820 in LUAD, LIHC, and KICH (**Figure S6C**). SEMA3A has multiple identical methylation sites (cg03080692, cg16346212, cg23623142, cg26908950, cg10035469, cg15035350, cg05742308, cg20955022, cg06144675) in LUAD and LUSC (**Figure S6D**).

We also used the MethSurv database and generated heatmaps to identify differential methylation regions related to the predicted target genes. The highest methylation levels were found at cg000303876 of HNRNPAB, cg25750408 of PLAUR, and cg19762801/cg02271707/cg12998599 of SEMA3A (**Figure S7A**). Moreover, all SEMA3A-related methylation sites were located in the region of ‘open sea’ (**Figure S7A**). The expression of HNRNPAB/PLAUR/SEMA3A was significantly negatively correlated with their methylation levels (**Figure S7B**). In addition, the results demonstrated that 2 CpGs of HNRNPAB (cg09567732 and cg17587934), cg15459820 of PLAUR and 4 CpGs (cg00922200, cg09943050, cg06144675 and cg12862820) of SEMA3A were associated with significant prognosis (**Figure S7C**).

### Genomic alterations of predicted target genes in LUAD

We used the cBioportal database to determine the types and frequencies of genomic alterations of EME1/HNRNPAB/PLAUR/SEMA3A in LUAD samples. The results showed that SEMA3A had a mutation frequency of 5% (**Figure S8A).** We used the ‘correlation analysis’ module in the cBioportal database and found the expressions and copy numbers of all genes were significantly positively correlated (P < 0.05) (**Figure S8B**). The types of gene mutations are shown in **Figure S8C**. Missense mutations were the main type of genetic alteration in EME1/HNRNPAB/PLAUR/SEMA3A. Furthermore, as shown in **Figure S8D**, EME1 was altered in LUAD (3.04%) with copy number amplification as the main type of genomic alteration (an alteration frequency of approximately 2.61%). Copy number amplification (2.17%) was also the main type of genomic alteration in HNRNPAB, with an alteration frequency of approximately 2.61%, which was the same as that of PLAUR (2.61%). However, mutations were the main type of genomic alterations in SEMA3A (4.78%) (an alteration frequency of 3.48%). Therefore, the genetic alterations of the predicted target genes among LUAD samples in the TCGA cohort were different.

### Correlation between the expression of predicted target genes and immune infiltration

The relationship between the abundance of tumour-infiltrating lymphocytes (TILs) and the expression of EME1/HNRNPAB/PLAUR/SEMA3A in pan-cancer and LUAD was demonstrated on heatmaps and lollipop graphs (**Figure S9**). The expression of EME1, HNRNPAB, and SEMA3A was significantly associated with Th2 cell infiltration (r = 0.580, 0.350, and 0.300, respectively). PLAUR expression was associated with the infiltration of neutrophils, Th1 cells, macrophages, and NK CD56dim cells (r = 0.429, 0.401, 0.395 and 0.354, respectively) (**Figure S9C**).

**Figure 10:**
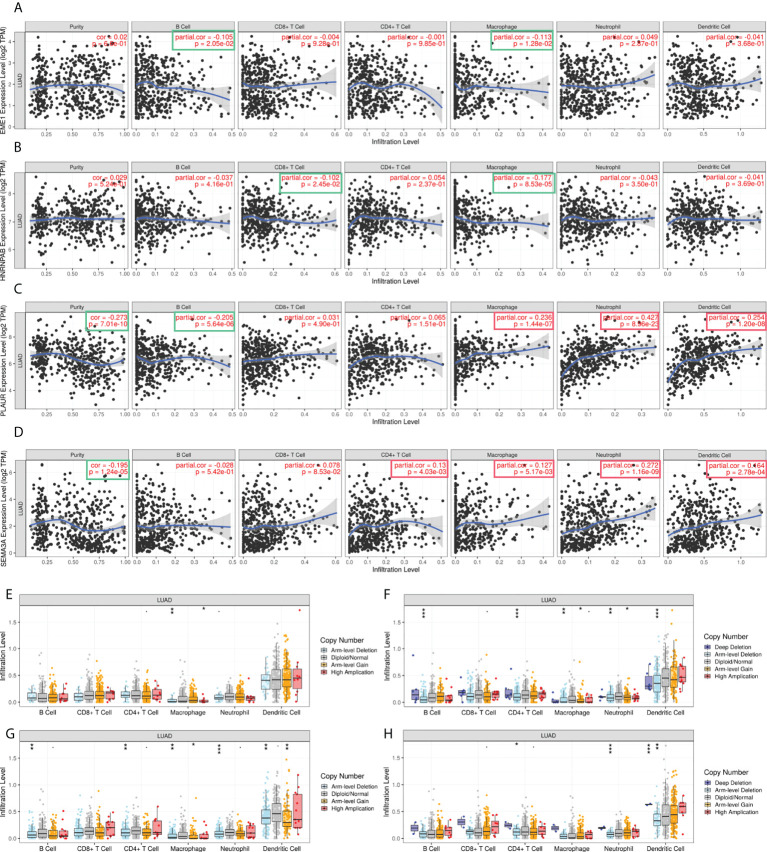
The correlation analysis of predicted target gene expression and immune infiltration. **(A–D)** Gene expression and immune infiltration level. **(E–H)** Gene copy number and immune cell infiltration level. *p < 0.05; **p < 0.01; ***p < 0.001.

In addition, TIMER was used to examine the relationship between the expression of EME1/HNRNPAB/PLAUR/SEMA3A and TILs in LUAD tissues. The results revealed that macrophages, neutrophils, and dendritic cells infiltration were positively correlated with the expression of PLAUR/SEMA3A (**Figures 10A–D**). Furthermore, the ‘SCNA’ module analysis showed that the arm-level deletion and high amplification levels of EME1 were significantly associated with CD4+ cell infiltration in LUAD (**Figure 10E**). The arm-level gain of HNRNPAB was significantly associated with the infiltration of macrophages and neutrophils (**Figure 10F**), whereas the arm-level deletion of HNRNPAB and PLAUR was significantly associated with the infiltration of B cells, CD4+ cells, macrophages, neutrophils, and dendritic cells (**Figure 10G**). The arm-level deletion of SEMA3A was significantly associated with the infiltration of CD4+ cells, neutrophils, and dendritic cells (**Figure 10H**).

### Functions of predicted target genes in LUAD cell lines

CCLE was used to assess the expression distribution of EME1/HNRNPAB/PLAUR/SEMA3A in pan-cancer cell lines (**Figure S10**). To investigate the underlying functions of the predicted target genes in LUAD, we performed single-cell analysis using the CancerSEA database (**Figure S11**) and found that EME1 negatively regulated inflammation in LUAD cells (**Figure S11)**, whereas HNRNPAB positively regulated cell cycle, stemness, invasion and proliferation in LUAD cells (**Figure S11**). In addition, both PLAUR and SEMA3A positively regulated metastasis, angiogenesis, quiescence, epithelial–mesenchymal transition (EMT), and differentiation in LUAD cells (**Figures S11C, D**).

### Expression of predicted target genes in lung tissues

**Figure 11:**
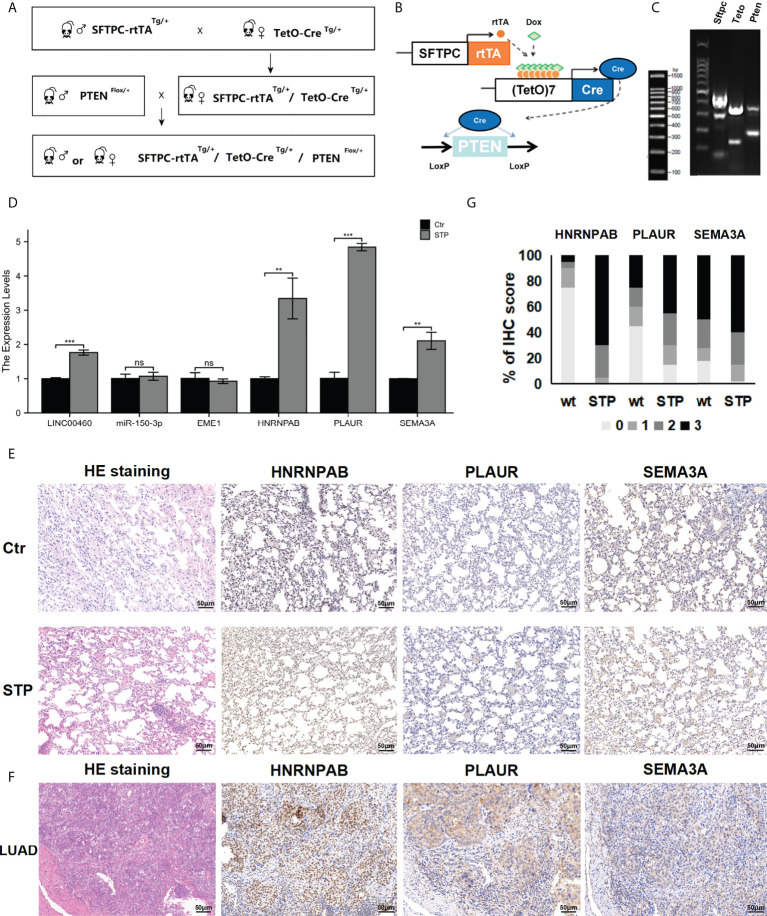
Expression of predicted target genes in lung tissue. **(A, B)** The construction of STP mouse model. **(C)** Genotyping results. **(D)** The expression of genes in PTEN-deficient lung tissues. **(E)** The HE and IHC staining results. **(F)** The expression of protein in PTEN-deficient lung tissues. **(G)** Percentage of malignant cells with different target genes IHC score in wt and STP groups. ns, no significant; *p < 0.05; **p < 0.01; ***p < 0.001.

To assessed the effect of PTEN deletion in LUAD development. We crossed conditionally PTEN-null mice (PTENflox/+) with lung-specific (SFTPC-rtTA/tetO-cre) mice. We generated lung epithelial-specific PTEN knockout mice (SFTPC-rtTA/tetO-cre/PTENflox/+; STP) (**Figures 11A–C**). The HE staining results showed that no macroscopic abnormality was observed in normal adult mice (age, 11 weeks) (**Figure 11E**). The lung tissue of STP mice was with early-stage alveolar epithelial hyperplasia and infiltration of inflammatory cells after 11 weeks (**Figure 11E**). However, other morphologic abnormalities were not found.

The expression of the mir-150-3p–LINC00460–EME1/HNRNPAB/PLAUR/SEMA3A axis in PTEN-deficient lung tissues was evaluated via qPCR analysis. According to the results, expressions of LINC00460/HNRNPAB/PLAUR/SEMA3A were significantly upregulated in the lung tissues of PTEN-deficient mice (**Figure 11D**). At the same time, HNRNPAB/PLAUR and SEMA3A levels were evaluated via IHC analysis. The results showed that HNRNPAB/PLAUR and SEMA3A were upregulated in the lung tissues of PTEN-deficient mice treated with doxycycline. However, EME1 expression was not significantly different between the STP group and the negative group (**Figure 11E**). We used a scoring system to consolidate the results and calculate the intensity and positive staining percentage of staining (**Figure 11G**). At the same time, the expressions of HNRNPAB/PLAUR and SEMA3A were detected in the cancer and paracancerous tissues of a lung adenocarcinoma patient, as determined by IHC. Expression of HNRNPAB was found to be up-regulated in lung cancer tissues compared with the cancer adjacent tissues (**Figure 11F**). For the control, HNRNPAB expression was expressed in the nucleus. The results also showed that the expression levels of PLAUR and SEMA3A were significantly upregulated in the lung cancer tissues (**Figure 11F**). For the control, PLAUR and SEMA3A were expressed in the cytoplasm.

### Cancer pathway activity and drug sensitivity analysis

**Figure 12:**
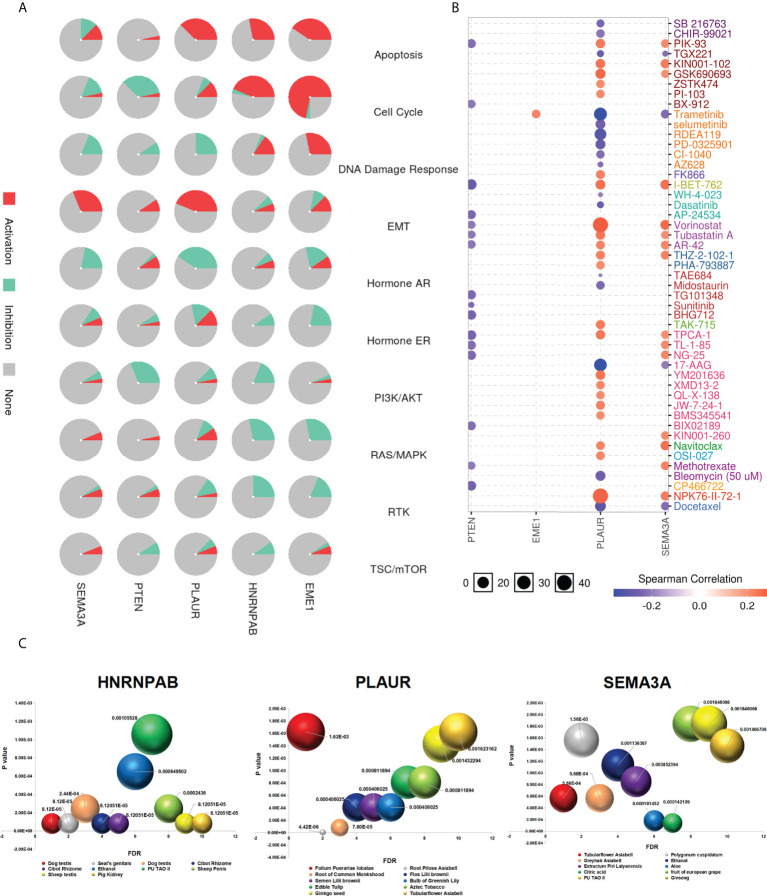
Drug sensitivity analysis. **(A)** The role of predicted target genes in cancer pathway activity in LUAD. **(B)** Drug sensitivity and gene expression data. **(C)** Chinese herbal medicines potentially target genes.

The GSCA database was used to examine the role of EME1/HNRNPAB/PLAUR/SEMA3A in 10 cancer pathways and found that EME1 was mainly involved in the activation of apoptosis, cell cycle and DNA damage response and inhibition of RAS/MAPK pathways. Similar results were observed in HNRNPAB. PLAUR was mainly involved in the activation of apoptosis and EMT pathways and inhibition of the hormone AR pathway. However, the role of SEMA3A in cancer pathway activity was not significant, except in the EMT pathway (**Figure 12A**).

We used GDSC and CTRP databases to evaluate the relationship between the expression of genes and the sensitivity of multiple drugs, which yielded different results. Cells with high expression of EME1, PLAUR, and SEMA3A were resistant to 1, 22, and 15 drugs, respectively. In addition, cells with high expression of PLAUR and SEMA3A were sensitive to drugs such as docetaxel, 17-AAG, and trametinib (**Figures 12B** and **S12A**).

### Traditional Chinese medicine targets predicted genes

With increasing in the incidence rate of cancer and side effects of chemotherapy, the use of phytochemicals for chemoprevention has emerged as a promising treatment approach for malignant tumours. Therefore, identifying effective antitumor drugs from plants has become a major focus area of pharmaceutical research. We used the ‘BenCaoZuJian’ database and found 11, 15, and 9 Chinese herbal medications potentially targeting HNRNPAB, PLAUR, and SEMA3A, respectively (**Figure 12C**).

In addition, we found plant-derived traditional Chinese medicine components targeting both HNRNPAB and SEMA3A, including 17 beta-estradiol, a natural compound exhibiting broad anti-cancer effects against lung cancer (**Figure S12B**). Triptolide may be a plant-derived traditional Chinese medicine component targeting PLAUR (**Figure S12B**). Moreover, we identified some diseases and phenotypes related to HNRNPAB/PLAUR/SEMA3A from the database, including non-small cell lung carcinoma (NSCLC), colorectal carcinoma (CRC) and LUAD (disorder) (**Figure S12C**). In addition, the relationship between drug sensitivity and EME1/PLAUR and SEMA3A mutations was examined using the CAMOIP online tool (**Figure S12D**).

## Discussion

Identifying potential biomarkers and therapeutic targets are essential to improve the prognosis of lung adenocarcinoma (37). New strategies are urgently required to detect and prevent LUAD at an early stage. Loss of PTEN expression is frequently found in various cancers, including lung cancer (38). PTEN function may determine the outcome of lung cancer treatment (39). Recently, the ceRNA hypothesis has improved the understanding of oncogenesis (40). Increasing evidence has shown that lncRNAs and miRNAs are crucial in LUAD (41). However, clinical trials or targeted therapeutic drugs regarding PTEN have not been established in lung adenocarcinoma. Accordingly, in this study, we attempted to establish a PTEN-related ceRNA triple network in LUAD.

In the present study, we established a PTEN-related ceRNA triple network of LUAD, identifying 298 DElncRNAs, 188 DEmiRNAs, and 1527 DEmRNAs. The top 100 most relevant DERNAs were identified by the Cytoscape software. Furthermore, the correlation between the expression of genes in the hub triple regulatory network and the OS of patients were assessed. The DERNAs in the ceRNA network were analyzed through Cox regression, methylation, and immune infiltration analyses. In addition, we constructed a transgenic lung-specific conditional PTEN knockdown mouse model (SFTPC-rtTA/tetO-cre/PTENflox/+; STP). Changes in the expression of predicted target genes in lung tissues of STP mice were characterized by IHC analysis and qPCR. Eventually, we obtained the novel LINC00460–miR-150-3p–EME1/HNRNPAB/PLAUR/SEMA3A axis associated with the prognosis of LUAD.

Recently, many studies have analyzed DElncRNAs in normal and LUAD tissues and have shown that LINC00460 is closely related to the progression of many types of diseases including cancer, which may become a new therapeutic strategy for EGFR-mutant LUAD (42). Another study identified that LINC00460 could promote the progression of LUAD by regulating miR-302c-5p (43). However, the specific mechanisms of LINC00460 remain unclear. Therefore, we speculated that LINC00460 could exert its biological function as a ceRNA during LUAD progression. We examined the association between the expression of the LINC00460/mir-150-3p axis and OS in patients with LUAD to verify its prognostic potential and found that high expression of LINC00460 indicated a worse prognosis. In addition, the expression of LINC00460 was significantly associated with ‘age’ and ‘smoking status of patients (P < 0.05). Therefore, these results indicated that LINC00460 acts as an oncogene in the progression of LUAD.

It is well known that the binding of miRNAs to lncRNAs can reduce miRNA levels, resulting in an increased expression of miRNA target genes (44). Several studies have reported that certain miRNAs can directly target PTEN (45). We used the miRcode database to screen for DEmiRNAs interacting with LINC00460 and observed that miR-150-3p closely interacted with LINC00460. Studies have confirmed that miR-150-3p is downregulated in several types of cancers and acts as an anti-tumour miRNA (46). Similar effects of miR-150 have been found in other cancers, such as liver cancer, esophageal cancer, prostate cancer, and NSCLC (47). In this study, we found that the expression of mir-150-3p was downregulated in the PTENhigh expression group (P < 0.05), and the expression of LINC00460 and mir-150-3p was negatively correlated. In addition, high mir-150-3p expression was associated with a good prognosis in patients with LUAD. Therefore, we speculated that LINC00460 promoted the progression of LUAD by negatively regulating mir-150-3p.

Furthermore, we analyzed the expression of ceRNAs in tumour and control samples using TCGA, GTEx, and CPTAC databases and found that the expressions of LINC00460/EME1/HNRNPAB/PLAUR/SEMA3A were significantly higher in LUAD tissues. In addition, the expression of mir-150-3p was significantly lower in LUAD tissues than in the corresponding normal tissues. Moreover, the expression of ceRNAs in most pan-cancer tissues showed similar results, which further supported our hypothesis. The enrichment analysis revealed that most pathways enriched by PLAUR- and SEMA3A-related genes were duplicated, suggesting that these two genes are involved in the same pathways leading to cancer development. Gene and protein interaction analysis revealed that EME1/HNRNPAB/PLAUR/SEMA3A and PTEN were co-expressed with TP53. In addition, EGFR was identified as one of the potential target proteins of PLAUR. Similarly, the correlation of PLAUR expression was significantly associated with the EGFR expression in LUAD. The expression of SEMA3A was significantly associated with EGFR and PD-L1 expression in LUAD. Our results revealed that there is a potential correlation between EGFR/PD-L1 and the PTEN-associated ceRNA regulatory network.

Studies have shown that abnormalities in DNA methylation are related to cancer and many other diseases (48). In this study, we found that HNRNPAB and PLAUR were hypomethylated in LUAD samples compared with control samples, which was consistent with their upregulated expression in LUAD tissues. Therefore, we used several databases to explore possible explanations for the abnormal expression of these predicted target genes at DNA methylation levels in LUAD. According to the MEXPRESS database, the methylation of HNRNPAB/PLAUR/SEMA3A was associated with several clinical factors including ‘new tumour event after initial treatment’ and ‘tumour stage’ (**Figure 9C**). In addition, the clinical factors ‘the number of pack years smoked’ and ‘cigarettes smoked per day’ were significantly associated with EME1 methylation. We used the methylation module of the cBioPortal database to evaluate the correlation between methylation and expression of predicted target genes in LUAD samples. According to the data, the expression of HNRNPAB/PLAUR/SEMA3A was significantly negatively correlated with the DNA methylation level (**Figure S7B**). Therefore, abnormal methylation of predicted target genes may be associated with the poor prognosis of LUAD. The results demonstrated that the CpG sites cg15459820 of PLAUR and cg06144675 of SEMA3A were associated with significant prognosis. Most importantly, these two methylation sites also occur in other cancers and require further study (**Figure S6**).

TILs are evidence of an immune response against malignant tumours (49). LUAD is one of the cancers with the highest level of immune infiltration, especially T cell infiltration (50). In the present study, we observed that the expression of EME1, HNRNPAB, and SEMA3A was significantly associated with Th2 cell infiltration. In addition, PLAUR expression was associated with the infiltration of neutrophils, Th1 cells, macrophages, and NK CD56dim cells. TIMER analysis was performed to examine the relationship between the expression of predicted target genes and TILs in LUAD. ‘SCNA’ module analysis revealed that the arm-level deletion of HNRNPAB and PLAUR was significantly associated with the infiltration of B cells, CD4+, macrophages, neutrophils and dendritic cells. The results suggest that EME1, HNRNPAB, PLAUR, and SEMA3A serve as crucial immuno-modulation factors in LUAD. These findings suggest that changes in the tumor immune microenvironment and the development of LUAD may be influenced by the LINC00460-mir-150-3p axis and its downstream target genes.

The expression of predicted target genes in lung tissue was evaluated via conditional deletion of PTEN in mice. By crossing PTENflox/+ mice with SFTPC-rtTA/tetO-cre mice, to specifically inactivate PTEN in the lung tissue, we constructed an SFTPC-rtTA/tetO-cre/PTENflox/+ (STP) mouse model. Furthermore, we determined the localization and expression of HNRNPAB, PLAUR, and SEMA3A in the lung tissues of STP mice via qPCR and IHC analysis and found that HNRNPAB, PLAUR, and SEMA3A were upregulated in PTEN-deficient mice. IHC staining also confirmed that HNRNPAB, PLAUR, and SEMA3A protein expressions in lung cancer specimens were significantly increased, which supported our hypothesis. The HNRNPAB gene belongs to a family of ubiquitously expressed heterogeneous nuclear ribonucleoproteins (hnRNPs) (51). HNRNPs play an important role in the pathogenesis of various cancers (52). However, the expression pattern of HNRNPAB in lung cancer have not been reported (53). In this study, the expression of HNRNPAB was significantly higher in LUAD tissues than in the corresponding control tissues. Changes in the expression of HNRNPAB are correlated with the immune infiltration of LUAD. However, the specific mechanisms underlying the role of HNRNPAB in LUAD require further investigation. PLAUR has been reported to exert antitumor effects (54). Studies have reported that PLAUR may be a new therapeutic target for gefitinib-resistant NSCLC (55). We found that the expression of PLAUR was significantly different in paired LUAD tissues. In addition, PLAUR expression was associated with the infiltration of multiple immune cells. Moreover, patients with high expression of PLAUR had poor OS. SEMA3A is one of class 3 semaphorins (56). However, the role of SEMA3A in LUAD is rarely reported. Therefore, this study may provide new therapeutic targets for LUAD. It is worth noting that we did not observe significant changes in tumor volume during the study. We believe that loss of PTEN may not directly result in statistically significant changes in tumor growth (57). This discrepancy may be due to the different mouse genetic backgrounds (58). The difference may be also related to the timing of sample collection. Other factors may also be important despite not being identified in our analyses. Further research exploring these and other possible explanations for the findings is needed.

To date, surgery, radiotherapy, and chemotherapy are the main treatment modalities for lung cancer (59). Recently, the development of drugs targeting specific genetics has greatly improved the treatment of patients with LUAD (60). Despite significant progress, there is a requirement for new effective diagnostic tools and potential drug targets for LUAD. Therefore, we speculate that the oncogenes associated with PTEN identified in this study are promising targets for the development of novel targeted drugs for LUAD treatment. We integrated profiling data from two databases, GDSC and CTRP, and evaluated the relationship between the expression of predicted target genes and multi-drug sensitivity and found that high expression of EME1, PLAUR and SEMA3A conferred resistance to 1, 22 and 15 drugs, respectively. In addition, cells with high expression of PLAUR/SEMA3A were sensitive to drugs such as docetaxel, 17-AAG, and trametinib. The side effects of radiotherapy and chemotherapy cannot be ignored. Therefore, it is of great practical significance to identify novel anti-tumour drugs with low toxicity and high potency for targeting tumour cells. We also analyzed the BenCaoZuJian database and found that several Chinese herbal medications potentially target HNRNPAB/PLAUR and SEMA3A (animal- and plant-derived drugs, respectively). However, no data was available for EME1 in the dataset. In addition, we found plant-derived traditional Chinese medicine components targeting both HNRNPAB and SEMA3A, including 17 beta-estradiol, a natural compound exhibiting broad anti-cancer effects against lung cancer. Triptolide may be a plant-derived traditional Chinese medicine component targeting PLAUR. Moreover, we found that diseases and phenotypes such as LUAD and NSCLC are associated with HNRNPAB, PLAUR, and SEMA3A. Therefore, the identification of drugs targeting predicted genes provides a rationale for improving the treatment of LUAD. However, this study has several limitations. Although the expression levels between predicted genes and PTEN were significantly correlated, a strong correlation between the LINC00460/miR-150-3p axis and downstream target genes was not observed as expected (**Figure S14**). The mechanisms underlying the oncogenic roles of the target genes in LUAD remain unknown and should be confirmed in future studies. In addition, LINC00460 and other potential targets of miR-613 warrant further investigation.

Furthermore, HNRNPAB/PLAUR and SEMA3A were considered by us as significant predictive target genes of the PTEN-related LINC00460/miR-150-3p axis in lung adenocarcinoma. The PTEN-related ceRNA network established in this study was significantly correlated with clinical prognosis, methylation, and immune cell infiltration. IHC analysis and qPCR analysis confirmed the upregulation of predicted target genes in PTEN-deficient lung tissues. Besides, we identified chemotherapeutic drugs and traditional Chinese medications associated with predicted target genes from the database. Therefore, our study helps to improve the understanding of the involvement of PTEN-related ceRNA networks in the carcinogenesis of lung cancer, the infiltration of immune cells in cancer tissues, and the evolution and prognosis of LUAD. In conclusion, our study provides a theoretical basis for the research and development of PTEN-related therapeutic drugs for tumours.

## Data availability statement

The datasets presented in this study can be found in online repositories. The names of the repository/repositories and accession number(s) can be found in the article/**Supplementary Material**.

## Ethics statement

All experimental procedures were approved by the Institutional Animal Care and Use Committee (IACUC) guidelines at Tongji University School of Medicine (SYDW-19-215). Written informed consent was obtained from the individual(s) for the publication of any potentially identifiable images or data included in this article.

## Author contributions

RX, G-XJ, Y-SM, and DF designed the study. J-BL, SL, and L-KH contributed to data analysis, drafting, or revising the article. WW, C-YJ, and C-YW performed the statistical analysis. RX, BS, and Y-JJ contributed equally to this work. All authors contributed to the article and approved the submitted version.

## Funding

This study was supported partly by grants from Natural Science Foundation of Jiangsu Province (BK20201442), Shanghai Natural Science Foundation (20ZR1472400), Key Program of Hunan Provincial Department of Science and Technology (2020WK2020).

## Acknowledgments

We would like to thank Dr. Wen Li for data analysis and critical discussion of the manuscript. We thank Bullet Edits Limited for the linguistic editing and proofreading of the manuscript.

## Conflict of interest

The authors declare that the research was conducted in the absence of any commercial or financial relationships that could be construed as a potential conflict of interest.

## Publisher’s note

All claims expressed in this article are solely those of the authors and do not necessarily represent those of their affiliated organizations, or those of the publisher, the editors and the reviewers. Any product that may be evaluated in this article, or claim that may be made by its manufacturer, is not guaranteed or endorsed by the publisher.
