# Supplementary material for: Comprehensive analysis to identify a novel PTEN-associated ceRNA regulatory network as a prognostic biomarker for lung adenocarcinoma
Source: Front Oncol. 2022 Aug 24;12:923026. doi: 10.3389/fonc.2022.923026 (PMC9449356; doi:10.3389/fonc.2022.923026)
Supplement: Supplementary file 15 [file Table_1.docx]

**Table S1. The PTEN expression in pan-cancer of TCGA samples.**

| **Cancer Type** | **Tumor/Normal** | **Number of samples** |
| --- | --- | --- |
| ACC | Tumor | 79 |
| BLCA | Normal | 19 |
| BLCA | Tumor | 414 |
| BRCA | Normal | 113 |
| BRCA | Tumor | 1109 |
| CESC | Normal | 3 |
| CESC | Tumor | 306 |
| CHOL | Normal | 9 |
| CHOL | Tumor | 36 |
| COAD | Normal | 41 |
| COAD | Tumor | 480 |
| DLBC | Tumor | 48 |
| ESCA | Normal | 11 |
| ESCA | Tumor | 162 |
| GBM | Normal | 5 |
| GBM | Tumor | 169 |
| HNSC | Normal | 44 |
| HNSC | Tumor | 502 |
| KICH | Normal | 24 |
| KICH | Tumor | 65 |
| KIRC | Normal | 72 |
| KIRC | Tumor | 539 |
| KIRP | Normal | 32 |
| KIRP | Tumor | 289 |
| LAML | Tumor | 151 |
| LGG | Tumor | 529 |
| LIHC | Normal | 50 |
| LIHC | Tumor | 374 |
| **LUAD** | **Normal** | **59** |
| **LUAD** | **Tumor** | **533** |
| LUSC | Normal | 49 |
| LUSC | Tumor | 502 |
| MESO | Tumor | 86 |
| OV | Tumor | 379 |
| PAAD | Normal | 4 |
| PAAD | Tumor | 178 |
| PCPG | Normal | 3 |
| PCPG | Tumor | 183 |
| PRAD | Normal | 52 |
| PRAD | Tumor | 499 |
| READ | Normal | 10 |
| READ | Tumor | 167 |
| SARC | Normal | 2 |
| SARC | Tumor | 263 |
| SKCM | Normal | 1 |
| SKCM | Tumor | 471 |
| STAD | Normal | 32 |
| STAD | Tumor | 375 |
| TGCT | Tumor | 156 |
| THCA | Normal | 58 |
| THCA | Tumor | 510 |
| THYM | Normal | 2 |
| THYM | Tumor | 119 |
| UCEC | Normal | 35 |
| UCEC | Tumor | 552 |
| UCS | Tumor | 56 |
| UVM | Tumor | 80 |

**Table S2. IHC of PTEN in Human Protein Atlas database.**

| **No.** | **Tissue type** | **ID** | **Age** | **Gender** | **staining** |
| --- | --- | --- | --- | --- | --- |
| **1** | Lung normal tissue | 1470 | 65 | male | low |
| **2** | Lung normal tissue | 2208 | 67 | female | low |
| **3** | Lung adenocarcinoma | 1327 | 54 | male | not detected |
| **4** | Lung adenocarcinoma | 4869 | 42 | female | not detected |

**Table S3. A list of primers used in qPCR analysis.**

| **Gene** | **Forward primer(5’-3’)** | **Reverse primer(5’-3’)** |
| --- | --- | --- |
| **LINC00460** | TCGGCTAAGAGTCACCCTGGATG | CACAGACGCCTCCCACACAATG |
| **mir-150-3p** | AAGAAGACTGGTACAGGCCTG | CAGTGCAGGGTCCGAGGT |
| **EME1** | CACTTGGAGGAGGAGGAGGACAG | GCCAGGACCAACACCAGGATTG |
| **HNRNPAB** | ATCCCAAGTTGAACAAAAGACG | CATACTGCTGTTGCTGATACAC |
| **PLAUR** | GAACAATACCCTTGGGTGTTC | CAGCCTCTTACGGTATAACTCC |
| **SEMA3A** | CATGCTCACGCTATTTTCCTAC | GATGATTATCATGGTGCTGCAA |

**Table S4. Univariate and multivariate analyses of clinicopathological characteristics among LUAD patients.**

| **Characteristics** |  | **Univariate Cox** |  |  | **Multivariate Cox** |  |
| --- | --- | --- | --- | --- | --- | --- |
|  | **HR** | **95% CI** | **P value** | **HR** | **95% CI** | **P value** |
| **TNM stage** | 2.623 | 1.925-3.575 | **0.001*** |  |  |  |
| **Diameter** | 2.367 | 1.623-3.452 | **0.001*** | 2.108 | 1.441-3.083 | **0.001*** |
| **Lymph-node metastasis** | 2.605 | 1.939-3.501 | **0.001*** | 2.398 | 1.774-3.243 | **0.001*** |
| **Distant metastasis** | 1.168 | 0.816-1.674 | 0.396 |  |  |  |
| **EME1**  **expression** | 1.502 | 1.121-2.014 | **0.007*** | 1.370 | 1.013-1.854 | **0.041*** |

* values indicate P < 0.05.

**Table S5. Univariate and multivariate analyses of clinicopathological characteristics among LUAD patients.**

| **Characteristics** |  | **Univariate Cox** |  |  | **Multivariate Cox** |  |
| --- | --- | --- | --- | --- | --- | --- |
|  | **HR** | **95% CI** | **P value** | **HR** | **95% CI** | **P value** |
| **TNM stage** | 2.623 | 1.925-3.575 | **0.001*** |  |  |  |
| **Diameter** | 2.367 | 1.623-3.452 | **0.001*** | 2.090 | 1.429-3.057 | **0.001*** |
| **Lymph-node metastasis** | 2.605 | 1.939-3.501 | **0.001*** | 2.503 | 1.856-3.376 | **0.001*** |
| **Distant metastasis** | 1.168 | 0.816-1.674 | 0.396 |  |  |  |
| **HNRNPAB**  **expression** | 1.383 | 1.031-1.856 | **0.031*** |  |  |  |

* values indicate P < 0.05.

**Table S6. Univariate and multivariate analyses of clinicopathological characteristics among LUAD patients.**

| **Characteristics** |  | **Univariate Cox** |  |  | **Multivariate Cox** |  |
| --- | --- | --- | --- | --- | --- | --- |
|  | **HR** | **95% CI** | **P value** | **HR** | **95% CI** | **P value** |
| **TNM stage** | 2.623 | 1.925-3.575 | **0.001*** |  |  |  |
| **Diameter** | 2.367 | 1.623-3.452 | **0.001*** | 2.003 | 1.367-2.935 | **0.001*** |
| **Lymph-node metastasis** | 2.605 | 1.939-3.501 | **0.001*** | 2.360 | 1.744-3.195 | **0.001*** |
| **Distant metastasis** | 1.168 | 0.816-1.674 | 0.396 |  |  |  |
| **PLAUR**  **expression** | 1.742 | 1.294-2.345 | **0.001*** | 1.489 | 1.095-2.024 | **0.011*** |

* values indicate P < 0.05.

**Table S7. Univariate and multivariate analyses of clinicopathological characteristics among LUAD patients.**

| **Characteristics** |  | **Univariate Cox** |  |  | **Multivariate Cox** |  |
| --- | --- | --- | --- | --- | --- | --- |
|  | **HR** | **95% CI** | **P value** | **HR** | **95% CI** | **P value** |
| **TNM stage** | 2.623 | 1.925-3.575 | **0.001*** |  |  |  |
| **Diameter** | 2.367 | 1.623-3.452 | **0.001*** | 2.090 | 1.429-3.057 | **0.001*** |
| **Lymph-node metastasis** | 2.605 | 1.939-3.501 | **0.001*** | 2.503 | 1.856-3.376 | **0.001*** |
| **Distant metastasis** | 1.168 | 0.816-1.674 | 0.396 |  |  |  |
| **SEMA3A**  **expression** | 1.412 | 1.053-1.892 | **0.021*** |  |  |  |

* values indicate P < 0.05.

**Table S8. Univariate and multivariate analyses of clinicopathological characteristics among LUAD patients.**

| **Characteristics** |  | **Univariate Cox** |  |  | **Multivariate Cox** |  |
| --- | --- | --- | --- | --- | --- | --- |
|  | **HR** | **95% CI** | **P value** | **HR** | **95% CI** | **P value** |
| **TNM stage** | 2.623 | 1.925-3.575 | **0.001*** |  |  |  |
| **Diameter** | 2.367 | 1.623-3.452 | **0.001*** | 2.090 | 1.429-3.057 | **0.001*** |
| **Lymph-node metastasis** | 2.605 | 1.939-3.501 | **0.001*** | 2.503 | 1.856-3.376 | **0.001*** |
| **Distant metastasis** | 1.168 | 0.816-1.674 | 0.396 |  |  |  |
| **LINC00460**  **expression** | 1.118 | 0.836-1.496 | 0.451 |  |  |  |

* values indicate P < 0.05.
